# Patient-partner engagement at the Centre de recherche du CHUS in the Province of Québec, Canada: from an intuitive methodology to outreach after three years of implementation

**DOI:** 10.1186/s40900-021-00258-1

**Published:** 2021-03-16

**Authors:** Denis Boutin, Susan C. Mastine, Luc Beaubien, Maryse Berthiaume, Denise Boilard, Jaime Borja, Edouard Botton, Janie Boulianne-Gref, Sylvie Breton, Christian-Alexandre Castellano, Gisèle Charpentier, Francois-Pierre Counil, Marie-Josée Cozmano, Pierre Dagenais, Guy Drouin, Marie-Josée Fortier, Caroline Francoeur, Louise Gagné, David Héraud, Denise Hêtu, Marie-Pier Houde, Ginette Ladouceur, Marjolaine Landry, Elisabeth Leblanc, Christine Loignon, Valéry Lussier, Annie Morin, Nathalie Ouellet, Claude Quintin, Avinash Ramnarine, Catherine Wilhelmy, Amy Svotelis, Marie-Ève Thibault, William D. Fraser, Marie-Claude Battista

**Affiliations:** 1 Centre de recherche du CHUS, Sherbrooke, Québec Canada; 2 Direction de la coordination de la mission universitaire, Centre Intégré Universitaire de Santé et des Services Sociaux de l’Estrie – Centre Hospitalier Universitaire de Sherbrooke, Sherbrooke, Québec Canada; 3 Department of Pediatrics, Université de Sherbrooke, Sherbrooke, Québec Canada; 4 Department of Medicine, Rheumatology Division, Université de Sherbrooke, Sherbrooke, Québec Canada; 5 Faculty of Medicine and Health Sciences, Université de Sherbrooke, Sherbrooke, Québec Canada; 6 Department of Nursing, Université du Québec à Trois-Rivières, Trois-Rivières, Québec Canada; 7 Department of Surgery, Orthopedics Division, Université de Sherbrooke, Sherbrooke, Québec Canada; 8 Centre de recherche - Charles-Le Moyne – Saguenay – Lac-Saint-Jean sur les innovations en santé (CR-CSIS), Québec, Canada; 9 Department of Emergency and Family Medicine, Université de Sherbrooke, Sherbrooke, Québec Canada; 10 Department of Obstetrics and Gynecology, Faculty of Medicine and Health Sciences, Université de Sherbrooke, Sherbrooke, Québec Canada; 11 Department of Medicine, Faculty of Medicine and Health Sciences, Université de Sherbrooke, 3001, 12e Avenue Nord, Sherbrooke, Québec J1H 5N4 Canada

**Keywords:** Patient engagement, Research, Governance, Patient and public involvement, Partnership, Contribution, Patients networking

## Abstract

**Background:**

Medical societies and funding agencies strongly recommend that patients be included as partners in research publications and grant applications. Although this “top-down” approach is certainly efficient at forcing this new and desirable type of collaboration, our past experience demonstrated that it often results in an ambiguous relationship as not yet well integrated into the cultures of either patients’ or the researchers’. The question our group raised from this observation was: “How to generate a cultural shift toward a fruitful and long-lasting collaboration between patients and researchers? A “bottom-up” approach was key to our stakeholders. The overall objective was to build a trusting and bidirectional-ecosystem between patients and researchers. The specific objectives were to document: 1) the steps that led to the development of the first patient-partner strategic committee within a research center in the Province of Québec; 2) the committee’s achievements after 3 years.

**Methods:**

Eighteen volunteer members, 12 patient-partners and 6 clinician/institutional representatives, were invited to represent the six research themes of the Centre de recherche du CHU de Sherbrooke (CRCHUS) (Quebec, Canada). Information on the services offered by Committee was disseminated internally and to external partners. Committee members satisfaction was evaluated.

**Results:**

From May 2017 to April 2020, members attended 29 scheduled and 6 ad hoc meetings and contributed to activities requiring over 1000 h of volunteer time in 2018–2019 and 1907 h in the 2019–2020 period. The Committee’s implication spanned governance, expertise, and knowledge transfer in research. Participation in these activities increased annually at local, provincial, national and international levels. The Patient-Partner Committee collaborated with various local (*n* = 7), provincial (*n* = 6) and national (*n* = 4) partners. Member satisfaction with the Committee’s mandate and format was 100%.

**Conclusions:**

The CRCHUS co-constructed a Patient-Partner Strategic Committee which resulted in meaningful bilateral, trusting and fruitful collaborations between patients, researchers and partners. The “bottom-up” approach - envisioned and implemented by the Committee, where the expertise and the needs of patients complemented those of researchers, foundations, networks and decision-makers - is key to the success of a cultural shift. The CRCHUS Committee created a hub to develop the relevant intrinsic potential aimed at changing the socio-cultural environment of science.

## Plain English summary

In 2017, the Centre de recherche du Centre hospitalier Universitaire de Sherbrooke, a clinical research center affiliated with the Centre Intégré Universitaire de Santé et Services Sociaux de l’Estrie - Centre hospitalier Universitaire de Sherbrooke and the Université de Sherbrooke, strived to reconcile the institution’s decision-making/governance and research agenda standards with patients’ needs and interests. After benchmarking and based on our intuition of the effectiveness of a “bottom-up” approach and knowledge in patient engagement, our group implemented the Province of Québec’s first clinical research center patient-partner’s strategic committee (the Committee), which is constituted of 12 patients and 6 clinician researchers/representatives. The Committee’s strategies hinge on principles of trust and respect and on the group’s diversified expertise to guide the challenges associated with patient engagement in research.

The Committee members contributed almost 2000 h of volunteer work in the academic year 2019–2020. From May 2017 to April 2020, and through collaborations with 17 internal and external partners, Committee members contributed to 53 categories of activity spanning governance, expertise and knowledge transfer in research, which took place at the local, provincial or national levels.

The three-year period necessary to implement the Committee, to gain the scientific/researcher community to endorse its mission and to create a trustful environment with partners provided a strong proof-of-concept for the potential impact such an institutionally-embedded hub may exert in research. The next step will be to measure the long-term impact of the Committee’s actions to accelerate the discovery and implementation of health solutions, which is the target of both patients and their physicians.

## Background

Patient engagement in research is not an emerging field as it has been evolving over time. In the late 1980s, persons with acquired immune deficiency syndrome and breast cancer were among the first advocates of a more prominent place and active participation of patients in research [1]. Their pleas aroused the interest of some governments to implement initiatives designed to achieve these ends. Among them, the National Institute for Health Research-UK launched, in 1996, the INVOLVE program aimed at supporting and encouraging patient participation in research [2]. Fourteen years later, the Patient-Centered Outcome Research Institute (PCORI) was established in the US [3]. Early on, Canada implemented strategies to actively engage patients in research. The Canadian Institutes of Health Research (CIHR) published its *Strategy for Patient-Oriented Research (SPOR)* [4] in 2011 and three years later, posted its framework clarifying the role and importance of patients in research [5]. These landmark initiatives led to patient engagement becoming a new standard in research. Scientific societies in various fields of medicine have recommended that, whenever possible, research be conducted in close partnership with patients [6, 7].

Patient engagement in research is driven by the participants’ willingness and drive to enhance the evolution of research: “Nothing about us without us”, stated the BMJ [8]. According to this mantra, there are as many forms of participation and engagement as there are patient and researcher tandems. Although varied and innovative, roles and expectations must be defined as clearly as possible early on, from the initiation of the research project [9–12].

As patient involvement as research “collaborators” is important, their engagement at strategic levels, i.e. in decision-making of global research processes is also mandatory. In the Province of Québec, in 2015, the Université de Montréal supported the creation of the Centre of Excellence on Partnership with Patients and the Public (CEPPP). CEPPP was a pioneer in the conceptualization of patient engagement at all levels of the research process with a focus on shifting from a paternalistic approach to a more inclusive and cross-sectional interrelationship among stakeholders, including the patients. This was referred to as the Montreal Model [12]. In 2015, the CIHR-SPOR initiative implemented provincial SPOR SUPPORT Units all across Canada to better connect decision-makers and stakeholders with patients in order to better address their needs as well as to co-design evidence-based solutions [13].

While patients are the heart of clinical research, their first point of contact with research is a clinical research centre, where research projects are conducted and where patients are invited to contribute as participants. Accordingly, patient engagement has become a new standard in research, with the anticipation of efforts to implement concrete activities at the earliest stages of development. Research centres with clinical mandates are infrastructures of choice to implement and apply the concept of patient engagement. Clinical research institutions are at the cornerstone of patient needs and scientific breakthroughs. Among the functions of these health research centres conduct peer-reviewed competitions for their members through which funds are provided to generate preliminary results and proof-of-concept studies which serves as steppingstones to larger and national/international competitions. Accordingly, health research centers have the power to prioritize research ideas or create incentives to serve certain strategic orientations. These Centres also facilitate and provide services to ensure that clinical research be conducted according to rigorous international standards. Patients are thus at the heart of the mandates of these Centres. However, to our knowledge, as of May 2017, no patient committee was in place to advise a research centre’s board of directors or scientific council in the Province of Québec, nor in Canada. Such a gap is inconsistent with the mandate of publicly sponsored research organizations.

The Centre de recherche du Centre Hospitalier Universiaire de Sherbrooke (CRCHUS) is located in an urban area of the Province of Québec. Sponsored by the Fonds de recherche du Québec - Santé (FRQ-S) (the main research funding agency of the provincial government), the CRCHUS benefits from the expertise of 237 regular/active researchers (plus 53 associated members), provides supervision for nearly 740 students and postdoctoral fellows, all of whom contribute to the dynamism of the CRCHUS. Research activities in the CRCHUS’ focus on six themes, i.e. Cancer: Biology, prognosis and diagnosis (Cancer); Diabetes, Obesity and Cardiovascular Complications (DOCC); Medical Imaging (Imaging); Inflammation–Pain; Maternal and Child Health; Population Health: populations, organization, practices.

In 2017, while the concept of patient engagement was emerging, less than five CRCHUS-affiliated researchers were conducting research projects together with patients as collaborators, only two published manuscripts with patients as co-authors [14–18] and, at the level of governance, only the DOCC theme executive committee benefited from the input of patients. However, our limited local experience clearly indicated that fruitful and meaningful patient-partner collaborations were possible when backed by: 1) researchers who endorsed and acknowledged the added- and true-value of such a collaboration to their research program and; 2) dedicated patients who were not intimidated, nor paternalized by a clinician or scientist, and who were willing to contribute their experience to the benefit of science. These positive experiences led us to raise a red flag on why collaborations are relatively infrequent and when they do occur, often fail.

Based on these premises, in early 2017, the CRCHUS initiated a reflection on how to bridge these gaps. i.e. on how: 1) to promote both the quantity and quality of collaboration between researchers and patients; 2) to engage patients in the institution’s strategic decisions, and; 3) to develop strategies to facilitate the local, provincial, national and international dissemination of their initiatives. The key question that arose was: How to generate a cultural shift to gather, in a meaningful and sustainable way, decision-makers, researchers, research center staff and patients? The idea was to construct a hub for the development of patient engagement: a hub where all stakeholders could gather, discuss with an expert (based on theoretical or practical research knowledge or lived experience) and brainstorm on their questions. The purpose of this paper is to: 1) to document how our team guided the development of the Province’s first Health Research Centre Patient-Partner Strategic Committee; 2) to report its achievements after 3 years of activities (May 2017 to April 2020).

## Methods

### CRCHUS’s patient-partner strategic committee background and vision

In early 2017, the CRCHUS defined its vision of patient engagement following a thorough benchmarking of existing patient-oriented infrastructure in the Province of Québec, i.e. hospital users’ committees, initiatives in other Québec research centres, and the Sherbrooke Integrated University Health Network’s Patient-Partner Initiative Coordinator. To our knowledge, at that time, beside of the O’Brien Institute for Public Health at University of Calgary (Alberta) which had created the PaCER (Patient and Community Engagement research) initiative in 2008 to support relevant research projects development, no hospital based research centre had a patient-oriented committee in the province of Quebec to support research strategic decisions. In the spring of 2017, the decision to launch a Patient-Partner Committee was endorsed by the Scientific Council of the CRCHUS. A dedicated person was named to develop and lead the Committee (Institutional lead). The vision was to create a Patient-Partner Committee predominantly constituted of patients to: 1) develop a sustainable infrastructure in terms of patient engagement and equity; 2) build a sustainable model based on the local context.

### Committee membership

To achieve the objective associated with our vision, we determined that at least two patient representatives per theme would be involved allowing patient-members shared responsibility and flexibility in their attendance at meetings. This also favored collegiality, enabling discussions on common interests and objectives with a partner in the same theme. Patients were referred by local research coordinators and were briefly interviewed (15–30 min.) by the Institutional lead on their lived and research experience, as well as their motivation to join the Committee.

Eligibility criteria for Patient Committee members were: 1) aged 18 years or older; 2) active participation in a clinical research project in the past year; 3) no indication of a personal agenda; 4) not a CRCHUS employee. To reduce the burden on patients and to guide them in decision-making, three (3) CRCHUS affiliated clinician researchers (recruited based on interest) as well as three (3) institutional representatives (a research ethics advisor, the department head of CRCHUS infrastructure support team and the Institutional lead) were asked to join the Committee. These 18 representatives are voting Committee members.

The Patient-Partner Initiative Coordinator of the Université de Sherbrooke/Faculty of Medicine and Health Sciences’ (a group primarily focused on patient involvement in medical education) also sits on the Committee, as do patient-partners working in tandem with CRCHUS researchers. Although the status of these guests was “observers”, they were invited to participate in discussions at meetings, but were non-voting members. The Committee benefited from the support of an administrative assistant to assist and help coordinate Committee activities.

At the beginning of each year, the Patient-Partner Committee renewed its Executive Committee, which is constituted of a Chair, Vice-Chair, the Institutional lead and the administrative assistant. The Executive Committee was responsible for the oversight of the Committee’s mandates, decisions and preparation of the meetings. The Executive Committee was also responsible for follow-up and priority issues, communicating with external partners and preparing the Committee’s Annual Report, which was circulated to all members for their comments and approval and subsequently published on the Committee’s web page [19]. All members were required to sign a confidentiality agreement and a conflict-of-interest declaration.

The demographic composition of the Committee is described as follows: 1) 23 out of 32 current or past members were female; 2) Members were aged between 29 and 85 years; 3) Members were of diverse ethnic backgrounds (French Canadian, Latin American and European); and 4) One third of the members were retired from work, and the remaining were still actively employed citizens.

### Initial meetings

Subsequent to the committee’s launch in May 2017, the first six meetings enabled members to become acquainted with their colleagues, to better comprehend the research environment and scientific language and to better define, as a group, the Committee’s mandates. The Directors of each theme introduced their on-going activities as well as the facilitating factors and barriers their group faced in working with patient-partners. During these first meetings, the following items were discussed: members’ expectations; time, duration and frequency of meetings; financial compensation; roles, mandates, mission; quorum, voting issues, admissible expenses. In November 2017, a first version of the “Committee’s Governance Framework” was adopted by the Committee. This document was reviewed annually.

### Planning of meetings

Face to face meetings were scheduled 10 months in advance and the full year’s schedule was presented at the first meeting of each year. Based on a consensus, meetings were held on a monthly basis, starting in September and ending in June. With the goal of facilitating each member attendance, all members agreed that meetings would be scheduled between 6:30 pm to 8:30 pm.

Over the course of the month, requests and suggestions from internal/external partners or from members were addressed to the Committee lead and were compiled. Partners were invited to discuss their topics directly with committee members during meetings. Meetings, agendas, requests and related documents were discussed virtually with the Executive Committee members 1 week prior to each meeting. The meeting agenda and related documents were then transmitted to all members. The agenda for the meetings include general Committee information, decision, and action items as well as ethical issues (submitted by the ethics advisor member), news from monitoring\surveillance of the existing grey literature, review of the CRCHUS newsletter and a current events section.

During the meetings, all Committee members and observers were encouraged to participate based on the level of engagement that they were able to provide. Members were encouraged to frankly express their opinions during meetings but were not required to read or provide feedback on the documents provided. Members were not expected to share their personal health related experiences. Each meeting closed with a moment for members to voice their opinion on the conduct of the meeting, their future expectations and any final comments.

In addition to regular meetings, ad hoc working sessions and events were scheduled. These additional sessions were proposed to members and attended on a voluntary basis. Over the three-year period, members attended 29 scheduled and 6 ad hoc meetings or working session. Participation in training sessions and conferences were frequently planned for and offered to members. Members were invited to voluntarily participate in external committees or to attend external meetings requested by our partners.

### Costs related to the patient-partner committee’s activities

All Committee members volunteered their time. No member, including clinician and institutional representatives, was offered a compensation or a salary to prepare for or attend Committee meetings, aside from the administrative assistant. This condition was discussed annually, at the first meeting. Patient members were compensated for travel, parking and offered refreshments during meetings. This represented an annually $2000 (CAN) *in-cash* contribution shared by both the Sherbrooke Integrated University Health Network Patient-Partner Initiative and the CRCHUS. The CRCHUS assumed the cost of the salary of the Committee’s administrative assistant, whose tasks included preparing and attending the meetings as well as assisting the Executive Committee with its various mandates, representing a $9000 (CAN) *in-kind* contribution annually. When members attended external meetings or conferences, patients’ meeting-related fees were directly absorbed by the hosting party. Aside from preparing and attending Committee meetings, as a volunteer, the Institutional lead also represented the Committee at various meetings and events. When these meetings were scheduled during weekly working hours, a remuneration by the CRCHUS to the Institutional lead represented an annual investment of $15,000 (CAN). In 2017–2018, the Committee launched a patient-partner funding opportunity to CRCHUS researchers. The CRCHUS and the Sherbrooke Integrated University Health Network Patient-Partner Initiative each contributed a $5000 grant allowance for research projects – two projects were funded.

### Committee members’ satisfaction

In December 2019, Committee members completed a 10-question survey on their levels of satisfaction related to the format of the meetings, understanding of their roles and mandates of the Committee, understanding of the impact of achievements, degree of individual involvement, global appreciation and their individual prioritization of mandates. Twelve members completed the survey, eight of whom were patients and four of whom were institutional representatives (2 physicians, 2 coordinators). Committee members were invited to share 1–2 words best describing the implementation of a successful Patient-Partner Committee. This survey was generated in-house by the Executive Committee.

## Results

### Impact of the creation of the committee

The creation of the Committee generated a hub for patient-engagement which resulted in major impacts for the CRCHUS’s ecosystem. After three-years of activities, in 2020, 22 researchers engaged in different research activities together with 36 patient-partners. This is in opposition to the five researchers and the five patients that collaborated together in 2017, i.e. the year prior to the implementation of the Committee. The researcher-patient tandems either committed to develop new research ideas and projects, co-write scientific manuscripts or co-applied to peer-reviewed research competitions. Among these, one grant proposal was submitted and was funded by the Canadian Institute of Health Research (CIHR - Canada’s main research funding agency). The review panel recognized the contribution of the PP collaboration to the proposal. Indeed, engagement of patient-partner participation early on constitutes a major asset to the success of grant applications. Indeed, several of the grant proposals by CRCHUS researchers which were successful in the period between 2017 and 2020, included meaningful and fully engaged patient-partners, e.g. FRQS/Oncopole EMC^2^; New Frontiers in Research Fund/Government of Canada; CIHR-Project Grant; CIHR- Early Career investigator Grants in Maternal, Reproductive, Child & Youth Health. Although it is too soon to accurately measure the impact of including patient-partners on the number of publications, we are hopeful as two publications have been released between 2017 and 2020: A clinical practice guideline in the field of sepsis (BMJ 2018;362:k3284) and Preferences of patients with low back pain about non-surgical treatments (Patient Preference and Adherence 2019:13933–940).

In addition to the impact created on CRCHUS’s researchers, the Committee’s expertise and added-value extended rapidly to its ecosystem. The enthusiasm generated from the creation of the Committee led to the establishment of collaborations with different local (*n* = 7), provincial (*n* = 6) and national (*n* = 4) partners (Table 1).

**Table 1:** Committee partners

Partner acronym	Outreach	Definition of acronym/mission	Partner’s website
CRCHUS (and its 6 themes)^1^	Local	Centre for Research, Centre hospitalier universitaire de Sherbrooke	http://cr.chus.qc.ca/accueil/
IUPLSSS^2^	Local	Institut universitaire de première ligne en santé et services sociaux	https://www.iuplsss.ca/accueil/
PPI-FMHS^3^	Local	Patient-Partner Initiative - Faculty of Medicine and Health Sciences - Université de Sherbrooke	N/A
CIUSSSE-CHUS^4^	Local	Centre intégré universitaire de santé et de services sociaux de l’Estrie - Centre hospitalier universitaire de Sherbrooke	https://www.santeestrie.qc.ca/accueil/
FRQS – Oncopole^5^	Provincial	Fonds de recherche Québec - Santé	http://www.frqs.gouv.qc.ca/
McPeak-Sirois Group^6^	Provincial	Clinical Research in Breast Cancer	http://mcpeaksirois.org/en/
Q-CROC^7^	Provincial	Quebec-Clinical Research Organization in Cancer	https://qcroc.ca/en/
CIHR^9^	National	Canadian Institutes of Health Research	https://cihr-irsc.gc.ca/e/193.html
QBCF^10^	Provincial	Québec Breast Cancer Foundation	https://rubanrose.org/en
HSCTIAU^11^	Local	Health and Social Care Technology and Intervention Assessment Unit	https://www.santeestrie.qc.ca/professionnels/ressources-pour-les-professionnels/uetmisss/
DAC^12^	National	Diabetes Action Canada	https://diabestesaction.ca
Unité de SOUTIEN SRAP Quebec^13^	Provincial	Unité de SOUTIEN SRAP Quebec	http://unitesoutiensrapqc.ca/
CdRV^14^	Local	Research Centre on Aging	http://cdrv.csss-iugs.ca/home
Quebec Cancer Coalition^15^	Provincial	Quebec Cancer Coalition	http://coalitioncancer.com/en/
3CTN^16^	National	Canadian Cancer Clinical Trials Networks	https://3ctn.ca/page/patient-representative-advisory-committee
Colorectal Cancer Canada^17^	National	Colorectal Cancer Canada	https://www.colorectalcancercanada.com

Superscripts 1 to 17 are refered at Table 2

As the Committee was the first of its kind in the Province and as it gained attention, partners requested the advices and expertise of its members. Table 2 provides a full description of each activity conducted by the Patient-Partner Committee with partners. The Committee’s activities span four different categories, i.e. governance, expertise, research, and knowledge transfer.

**Table 2:** Patient-partner committee activities and patient’s implications

Outreach	Year	Type of activity	Partner / event	Description of patient’s implication
Local	2017	Governance	CRCHUS^1^	Development of the patient-partner’s strategic committee internal governance, conflicts of Interest and confidentiality frameworks.
Local	2017	Governance	IUPLSSS^2^, IPP-FMHS^3^	Consultation and guidance on the financial compensation vision and grid.
Local	2017-2018-2019	Expertise	Patient Partner Interinstitutional Committee (IUPLSSS^2^, IPP-FMHS^3^, CIUSSSE-CHUS^4^, CdRV^14^)	This Committee recognizes and improve patient-partners collaboration and engagement in research, healthcare management and training initiatives. In addition to the annual meeting organized by the group and where the PPSC presents its yearly achievement, working sessions are planned to share best practices and develop new tools.
Local	2017	Knowledge transfer (REB members)	CIUSSSE-CHUS^4^ Research Ethics Board	Training offered to the local REB board members on patient-engagement.
Local	2017-2018-2019	Knowledge transfer (scientific and web communities)	CRCHUS^1^	Conceptualization and updating of the PPSC’s web page. (https://www.crchus.ca/recherche-clinique/patients-partenaires/)
Local	2017-2018-2019	Knowledge transfer (scientific and web communities)	CRCHUS^1^	Decision-making on content of the CRCHUS’s monthly newsletter (https://www.crchus.ca/medias-publications/infolettre/).
Local	2018	Governance	CRCHUS^1^	Consultation and guidance on the development of the new CRCHUS branding strategy.
Local	2018	Expertise	CRCHUS^1^	Discussions with the CRCHUS’ theme directors on the PPSC’s contribution to the theme development and needs in terms of patient contribution (capacity building).
Local	2018	Research	CRCHUS^1^ IPP-FMSS^3^	Conceptualization and review of the first Patient-Partner Oriented internal research competition.
Local	2018	Knowledge transfer (physicians and medical residents)	CIUSSSE-CHUS^4^ Orthopedic Division, Annual Scientific Research Meeting	Presentation of the PPSC’s vision, mission, objectives in patient engagement in research.
Local	2019	Governance	CRCHUS^1^	Contribution to the 2020–2026 CRCHUS’s strategic plan for financial support from the provincial funding agency (FRQS). The PPSC collaborated to the development of the plan to increase patient engagement in research within the CRCHUS.
Local	2019	Governance	CRCHUS^1^ CIUSSSE-CHUS^4^	Consultation and guidance on financial compensation issues related to patient participation.
Local	2019	Expertise	CRCHUS^1^ - Cancer theme / Annual Symposium on Cancer Research, Inflammation-Pain theme / Annual scientific day; Population Health theme s / Annual scientific day; CIUSSSE-CHUS^4^ - Surgery Department / Annual scientific day	Oral presentations review committee - “Patient-partner award” award granted to the selected desserving graduate student presentation
Local	2019	Expertise	CRCHUS^1^ - Imaging theme ‘s annual scientific day	Presentation of the PPSC’s vision, mission, objectives and achievements on patient engagement and networking with researchers to establish partnerships.
Local	2019	Expertise	CIUSSSE-CHUS^4^ Research Ethic Board	Consultation and review of the CIUSSSE-CHUS biobank policy.
Local	2019	Expertise	CRCHUS^1^ Annual Retreat	Presentation of the PPSC’s vision, mission, objectives and achievements on patient engagement and networking with researchers to establish partnerships.
Local	2019	Expertise	CRCHUS^1^ - DOCC theme Annual scientific day	Networking with researchers to establish partnerships (collaboration between a patient-partner and a basic scientist emerged from this event)
Local	2019	Expertise	CRCHUS^1^Mother/Child theme Annual Retreat	Presentation of the PPSC’s vision, mission, objectives and achievements and networking with researchers to establish partnerships (collaboration between a patient-partner and a pediatric psychologist and the idea to develop a Youth Patient-Partner Committee emerged from this event)
Local	2019	Expertise	CIUSSSE-CHUS^4^ HSCTIAU^11^	Participation to the HSCTIAU advisory committee.
Local	2019	Expertise	CIUSSSE-CHUS^4^ - Musculoskeletal-pain healthcare trajectory	Participation to advisory board .
Local	2019	Research	CRCHUS^1^ - Mother/Child theme	Establishment of a researcher-patient collaboration to review and adapt the consent form and advice on the feasibility aspects of a cystic fibrosis research project.
Local	2019	Knowledge transfer (scientific, web, physicians, trainees, general audiences, decision-makers)	CIUSSSE-CHUS^4^	Conceptualization of the CIUSSSE-CHUS communication plan to promote patient-engagement internally and with external communities and partners
Local	2019	Knowledge transfer (general audience)	CRCHUS ^1^ – DOCC theme	Interviews to local media to promote and recognize patient engagement in research (print media, television, Facebook and radio) https://www.latribune.ca/actualites/sherbrooke/les-patients-partenaires-reconnus-comme-des-experts-7eb2fa54b9d0acc9730712db5cfbb9c1
Provincial	2018	Knowledge transfer (physicians, decision-makers, general audience with interests in breast cancer)	First Symposium on Breast Cancer Research of the McPeak-Sirois Group^6^	Invited speaker to share the PPSC’s vision, mission, objectives and achievements in patient-engagement and to network with researchers, partners and external partners (collaborations with GMPS, QBCF, Coalition Cancer and with breast cancer patients were initiated at this event).
Provincial	2018-2019	Expertise	Q-CROC^7^	Review of patient-oriented documents on clinical trials in oncology awareness (these documents were distributed across the Province of Québec).
Provincial	2018-2019	Knowledge transfer (general audience)	Q-CROC^7^	Hosting and participation at several booths across the Eastern Townships region healthcare centers - Clinical Research Awareness day campaign
Provincial	2019	Expertise	Quebec Cancer Coalition^15^	Advising on patient engagement in oncology
Provincial	2019	Expertise	Quebec Cancer Coalition^15^	Participation to the Annual Meeting, Annual Retreat, Day on political awareness of personalized medicine in oncology, Think Tank on clinical trial repositories, Community of practice “Experience and partnership in care and services”
Provincial	2019	Research	CRCHUS^1^ - Cancer theme	The All For One randomised controlled trial was co-designed with a breast cancer patient with a CRCHUS’s researcher (both member of the PPSC^2^). The idea was from the patient. The proposal was submitted for a CIHR catalyst grant (rejected) and a FRQS patient-priority (rejected) competitions. This project aims to design a patient-provided intervention to improve the quality of care offered to patients during the pre-diagnostic breast cancer period. This project is now being implemented at the national level in partnership with the QBCF.
Provincial	2019	Knowledge transfer (philanthropy partners)	Québec Breast Cancer Foundation^10^	Training offered to QBCF staff on patient engagement: barriers, facilitators and strategies to succeed.
Provincial	2019	Knowledge transfer (scientific patient partners)	Unité de SOUTIEN SRAP Québec ^13^	Member of the Patient Engagement Advisory Board - Contribution to the “Province of Quebec Health Research Patients and Citizen Partners Community of Practices”
Provincial	2020	Research	McPeak-Sirois Group^6^	Co-development of a proposal aimed at implementing a Provincial Breast Cancer Patient-Partner Committee, under the auspice of the GMPS, and local breast cancer patient-partners committees at GMPS participating institutions. The proposal was submitted to the “Quebec Breast Cancer Foundation Education Funds for Cancer $ 1 million initiative” (rejected). Currently, being implemented with the GMPS.
Provincial	2020	Research	FRQS-Oncopole^5^	Member of the Patient Priority’ competition Review Board, which mandate is to define national investment priorities and identify research projects likely to have a positive impact on cancer patients’ experience
National	2017	Expertise	Diabetes Action Canada^12^	The PPSC^2^ hosted the Diabetes Action Canada - Immigrant Committee Meeting and presented on the PPSC’s vision and objectives.
National	2018	Expertise	3CTN^16^	Member of the Patient Representative Advisory Committee and participation to the Annual Meeting.
National	2018	Research	CRCHUS^1^ - Mother/Child theme; Cancer theme; Population Health	Patient and researcher collaborations to research competitions: CIHR - Early Career Investigators in Maternal, Reproductive, Child & Youth Health - Operating Grants program (X3 tandem granted and patients named as co-investigators); CIHR - Project Grant Competition program (× 2 granted, patients named as collaborators); New Frontiers Research Funds - Exploration competition (× 1 granted, patients named as collaborators).
National	2019	Expertise	CIHR^9^	Participation to the CIHR public consultation on the “CIHR’s Ethical Guidelines for Establishing Research Partnerships with Patients” framework;
National	2019	Research	CRCHUS^1^	Member of Research Study Executive Committees: Epi-STORM (NCT03786991); WeCARE (NCT04254302); 3TMPO (NCT04000776); SOCRATIC (NCT04079764)
National	2019	Knowledge transfer (general audience)	Colorectal Cancer Canada^17^	Hosting and planning of the Giant Colon Tour booth. This event aimed to raise awareness on colorectal cancer and research.
Local Provincial National	2017-2018-2019	Research	CRCHUS^1^	The PPSC supports the CRCHUS’s investigators with letters of support and in-kind supports to local, national and provincial research grant competition applications. Emphasis is put on the supporting infrastructure developed in patient-oriented research to investigators and their patient-partners. In the 3 years covered herein, the PPSC has contributed to 15 letters of supports.
International	2019	Research	CRCHUS^1^ - Population Health theme CIUSSSE – CHUS^4^ - HSCTIAU ^11^	Contribution to peer-reviewed original papers on guidelines for sepsis (doi: 10.1136/bmj.k3284) and preference of patients with low back pain (doi: 10.2147/PPA.S201401)

Superscripts 1 to 17 refers to partners’ acronyms and missions, which are defined at Table 1

*Governance* refers to activities related to processes or high-level participation in the CRCHUS’s decision-making and efforts made to develop and expand patient engagement in research. As one example of the Committee’s implication in governance: together with the CRCHUS’s director, the Committee was invited to co-write the 2020–2026 CRCHUS Strategic Plan to receive its financial support from the provincial funding agency (FRQS). The Committee collaborated in the overall development of the plan by proposing strategies to increase patient engagement in research within the CRCHUS. At the time of external review, Committee members were invited to discuss with the FRQS external panel – a 1 h discussion was scheduled. The FRQS Committee included these remarks in their final report (translated from French): “*… the CRCHUS patient-partner’s initiative is very impressive and was much appreciated by our committee, …*”; “*The strategic patient-partner committee of the CRCHUS permits new opportunities for collaboration between members and users of the health system, which has and will generate new research ideas.*”; “*Important implication of patients in the different CRCHUS committees and decision-processes*”. The inclusion of patient-partners during this review process with FRQS certainly was seen as a major asset by the FRQ Committee to renew its confidence in the CRCHUS as an “excellent score” was provided and FRQS renewed its financing for the next 5 years.

*Expertise* refers to invitations to present as guest speakers, trainers, members of patient-partner external committees, reviewers, or appraisers. As examples, the Committee representative were invited to sit on the on board of the Patient Representative Advisory Committee of the Canadian Cancer Clinical Trials Networks (CCCTN). In addition, members were invited to review patient-oriented documents on clinical trials in oncology awareness, documents which are produced and distributed across the Province of Québec by Quebec-Clinical Research Organization in Cancer. Participation in these provincial and national committees provided patient-partners with the opportunity to impact on regional and national initiatives.

*Research* refers to significant contributions to the conception and co-design of research activities, such as project development, funding opportunity applications, original paper authorship and participation in clinical study’s Executive or Steering Committees. The integration of the Committee members on those clinical research committees positively impacts the feasibility of research projects and trials as they are better informed from inception by patients.

*Knowledge Transfer* refers to activities aimed at informing or transmitting information to different stakeholders, e.g. clinicians, decision-makers, patients. As an example of the Committee implication in this field, the McPeak-Sirois Group – a charitable organization and one of the most important breast cancer clinical research consortia in Canada – invited the Committee to present their work at the First Symposium on Breast Cancer Research, which they organized in the Sherbrooke area to inform community women. This activity led to a partnership with the McPeak-Sirois Group and the Committee is currently co-leading a project aimed at piloting breast cancer patient-partner committees at the different centers partnering with the McPeak-Sirois Group. As another example, through a partnership with the Centre d’excellence en neurosciences de Sherbrooke, the Committee contributed to the production of a theatre play in neurosciences (Neuro-Show) which will be presented in three main cities of the province of Québec (Sherbrooke, Montreal and Quebec) in the Spring of 2021. The participation of Committee members in these activities directly impacts our community as scientific knowledge is more widely disseminated, more accessible and understandable.

To achieve all the work and to contribute to the activities presented at Table 2, members committed over 1000 h of volunteer time to the Committee’s activities during the period from 2018 to 2019. Even though, as a result of the Covid-19 pandemic, the Executive Committee interrupted face to face meetings in April 2020 to ensure safety and health of Committee’s members and their families, all activities were pursued virtually. Therefore, even with the burden imposed by COVID-19, members volunteered over 1907 h between 2019 and 2020. Volunteer hours were not compiled for the year 2017–2018.

**Fig. 1:**
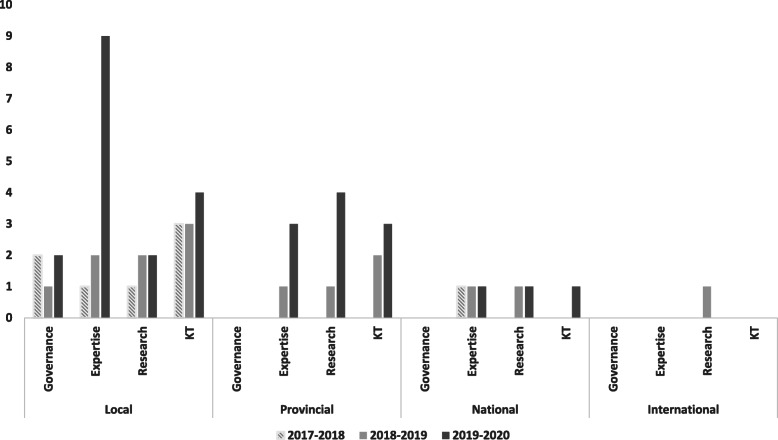
Number of activity types involving patient's engagement - Evolution by year and geographical outreach

In accordance with the increase in volunteer time is the concomitant increase in requests from partners. Figure 1 summarizes the number of activity types of the committee over the 2017–2018, 2018–2019 and 2019–2020 periods and their geographical scope (local, regional, provincial, national and international). Activities are represented by the number (discrete) of activity types rather than by number of contributions, e.g. patient-partner participation in the preparation of a peer-reviewed paper was counted as one activity type, while two contributions were made (refer to Table 2). Results presented in Figure 1 demonstrate that the Committee increased its contributions over the three periods and at the different geographical levels. Locally, the Committee increased its contributions from 7 activity types (governance =2; expertise = 1, research = 1, knowledge transfer =3) in 2017–2018 to 8 (governance =1; expertise = 2, research = 2, knowledge transfer =3) in 2018–2019 to 17 (governance =2; expertise = 9, research = 2, knowledge transfer = 4) in 2019–2020, with a total of 32 different participations in local activities over the three-year period.

The Committee did not contribute to any activity type at the provincial level in 2017–2018, whereas, in 2019–2020, it contributed to 10 activities, with a total of 14 events over the 2017–2020 period. At the national level, while only one activity type was noted in 2017–2018, there were 3 in 2019–2020, with a total of six contributions over 3 years. At the international level, only one activity type (peer-reviewed publications) was noted in the year 2018–2019. These trends were observable in the context of all four activity types (governance, expertise, research, knowledge transfer). This growth in activities was not formally planned, but rather organic. Although not validated through a formal survey assessing partner satisfaction, we hypothesize that this growth was secondary to the notoriety gained by the Committee over this three-year period as well as to the added-valued of the work performed by the Committee to each partners organization’ mission in relation to striving for patient’s input.

### Committee members’ satisfaction

Since the creation of the Committee, patient membership has been relatively constant slightly in composition, with only four voluntary departures due to either aging (*n* = 1) or health issues (*n* = 3, thus highlighting member’s loyalty and interest in maintaining their position and activities with the Committee. When members were questioned on the conditions required for a successful patient-partner committee, the following attributes were noted: respect, attentive listening, attribution of interest-based roles and the use of each member’s strengths, expectation management among members, common language, integrity, relevance, equity, authenticity, open communication and a unifying Institutional lead.

The results of the survey highlighted that 100% of respondents felt motivated by their participation in the Committee, appreciated the actual format of the meetings, understood the outreach the Committee exerted and felt that their contribution was real and made a difference. Results were similar when analysing all respondent responses as a whole or patient and clinical/institutional representatives separately. It was consensual from patient-partners that their participation in the Committee empowered them and increased their sense of meaningfully contributing to their community.

Patients highlighted that participation in the Committee changed their lives in many positive aspects: 1) it provided a sense to their living with the disease; 2) to contribute further; 3) give back what they have received.

The clinician/institutional members freely expressed the following:

“*As a research ethics advisor to both the researchers and the REB’s members, my role is to ensure that everyone adopts the best practices. One of my principal concerns is the protection of the participants. Being an institutional member of the Patient-Partner Committee makes me anticipate and increase my awareness to the issues faced by research participants. It allows me to better advise stakeholders. Also, I more frequently refer researchers to patient-partners when they encounter problems for which I’m not sure I have the right answer to.”*.

“*As an early career physician-researcher, the Committee gave me the opportunity to better understand the patients’ reality while participating in a study. It has shown to me how deeply interested and involved are our patients in improving our practice. Very refreshing*”.

“*Physicians / researchers participating in the Committee do not derive any academic benefit from their participation. The human experience of this participation and the possibility of contributing to the advancement of the cause, mainly in my area of ​​expertise in evaluation, constitute my own motivations*”.

« *My participation in this committee has been an occasion to expose myself to patients’ reality, perspectives and preferences outside the focussed context and interested relationships of research projects. I have been presenting research reports and papers produced in partnership with patients to my colleagues in order to promote this practice*”.

Members expressed, through the mean of the survey, the need to create working subcommittees to focus on important issues, namely governance and communication/dissemination. Seventy-five percent (75%) of respondents admitted that they would like to commit more to the committee’s activities on an individual basis. Sixty-seven percent (67%) of respondents highlighted that they would like to be more involved in the development of the committee’s governance – that they are now ready and have enough experience to develop a long-term vision. The following themes were prioritized by members: 1) pioneering in the development of patient engagement in the research environment; 2) involvement in the promotion and communication of research accomplishments; 3) involvement in designing research projects; 4) involvement in the strategic decisions of the institution; 5) involvement in the improvement of care offered to patients through research innovation; 6) involvement in developing support for researchers.

Accordingly, based on the results of this survey and on the increasing demand for consultation in the field of governance and communications/dissemination by partners, two sub-committees were created as of February 2020. Members were invited to join these two subcommittees according to their personal interests.

To develop a sustainable infrastructure that reflects its key stakeholders’ local strengths and shared vision, the Committee was initially co-constructed in action, i.e. based on the members evolving knowledge, expertise, experience and on their joy to work together as a group. To increase the Committee’ potential long-term impact it was envisioned to develop a sense of community and collaboration and to develop a clear and meaningful vision of the internal and partners’ needs prior to elaborating the Committee’s long-term mission, vision and value framework. The Governance Subcommittee works on the short- and long-term directions of the Committee and is committed to identifying key indicators of patient-partner’s impact in research. The Communications/Dissemination Subcommittee is pursuing two main objectives, i.e. the promotion of patient partnership and the demystification of research. They are developing material aligned with the governance subcommittee’s work.

### Satisfaction’s of the committee’s work by stakeholders

In recognition of the Committee’s overall achievements with patient-partners in oncology, the Committee was awarded, on November 22nd 2019, the 2019 Oncology Award (Inclusion of cancer patients category) from the Province of Québec’s Ministry of Health (Ministère de la Santé et des Services Sociaux) – Oncology Program for excellence. Although we did not formally surveyed our partners (which will be done in a forthcoming phase), the increase in the number of requests for partnership and contributing to more activities is an indication of their interests and appreciation (Fig. 1).

## Discussion

After 3 years of activities, the Committee co-constructed the Province of Québec’s first Research Centre Patient-Partner Committee using a bottom-up approach where patient-partners integrated a research institution, shared their vision with the director, researchers and local, provincial and national partners to nurture a cultural shift toward a fruitful and long-lasting collaborations in research. The multiple accomplishment by the Committee in the four different categories after only three-years of its launching is a first step, and a proof-of-concept, recognizing the need for such a hub/infrastructure in an ecosystem such as that of a health/medical research center as the CRCHUS. The key to the implementation of the Committee, its endorsement by its scientific community and the increase in true partnerships between stakeholders relied on three main factors: 1) the diversity of the forms of involvement in research of the Committee members, i.e. in overseeing governance processes, in sharing expertise, in co-constructing research projects and in disseminating results to various stakeholders; 2) working hand-in-hand with numerous partners (including researchers), who early on believed in the Committee’s potential, acknowledged its contribution and rapidly recognized the added-value of engaging patients; 3) the devoted volunteer engagement and enthusiasm of all members, who put their hearts and souls to the benefit of their communities.

The Patient-Partner Committee sought to better incorporate their vision with that of various stakeholders and to pave the way for additional patient-oriented research. To accomplish this goal, the Committee has been functioning at the crossroads of three different universes of stakeholders.

Firstly, patient members’ perspectives toward science, and scientific knowledge was addressed. Given that researchers acquired very specific expertise over a long and demanding academic journey, and that the amount of knowledge they acquired is vast, science may be perceived by patients to be impressive, hermetic and hardly understandable for people outside the community of scientists. A recent qualitative British study, aimed at exploring the relationship between researchers and patient partners in health sciences, highlighted that although much effort is invested to nurture a culture of merging scientific and patient perspectives, an unequal power dynamic often consciously or subconsciously arises. They underlined the need to better blend “scientific” with “experiential” knowledge [20, 21]. This is precisely the mission of our Patient-Partner Committee, i.e. to encourage researchers to partner with patients to facilitate the sharing of expertise and experience and to engage in projects, prioritized by patients, which will generate new knowledge and benefit as many patients and citizens as possible. Both scientists and patients must accept to engage on an unknown path and acknowledge each other’s genuine intentions. Scientists must develop this new reflex to consult patients and patients must develop confidence in the expertise they possess. To secure the conditions for collaboration to flourish, a group of philosophers, ethicists and sociologists has suggested that this type of participant-led research should be defined by a new social contract [22]. This is exactly what our Committee envisions with the co-construction of its governance framework.

Secondly, the Patient-Partner Committee incorporated decision-making structures to secure a bottom-up transmission of information so that health network administrators could play a role in developing research ecosystems favourable to the development of patient-centred knowledge. To attain this objective, health network administrators must believe in such an approach and promote patient input in research facilities and in the healthcare system. Indeed, patient input is mandatory and very relevant for health decision-makers [23]. The Province of Quebec’s Health and Well-Being Commissioner as well as the Commission on the Future of Health Care in Canada underlined the importance of hearing the citizen’s voice in the process of improving the management of care and services offered to patients [24, 25]. Health care administrators are striving for patient’s input. This is exactly what our Committee stands for. However, to attain this overarching objective, administrators must nominate institutional patient-partner initiative leader(s), who will develop an atmosphere of trust and respect, and they should also allocate financial resources to this end.

Thirdly, the Patient-Partner Committee served as a first opportunity to help patients realize that they can have a major impact on research development. Patients and caregivers who have struggled and dealt with health problems have gained knowledge throughout their life experience and in various living contexts. Furthermore, their “experiential” knowledge must be recognized as of great value and must be employed to benefit the society. It is now recognized that when patients are involved, research is more feasible to conduct, more acceptable to be implemented in the real world, more contextually based and more thought-provoking [26–28]. Patients must be incorporated as part of the research team; they must take their place: a place that should be opened by health network administrators and researchers. Along with other stakeholders, they must innovate in terms of the best ways to play a role in research and to be pioneers in defining patient experiential expertise.

Over the past 3 years, the Patient-Partner Committee created a hub to gather these stakeholders around patient engagement in research. Our knowledge of the existing literature suggests that while conceptual data exists suggesting the “why’s” and “how’s” to engage patients in research [29–33], sparse initiatives have published on the impact their patient engagement structure exerted on researchers, patients and the different stakeholders involved. Of these few initiatives, the PACER initiative, two patient advisory councils in Canada (Newfoundland/Labrador and Alberta [34], the Can-SOLVE CKD Research Operations Committee [35]. Manchester/England Public Programmes Team [36] must be mentioned as it proposes models of interdisciplinary public-practice oriented professionals to support public and patient engagement in health research. Although we agree with the authors that institutional leadership and the creation of a robust infrastructure are mandatory to secure long-term participation of patients in research, we strongly support that a “bottom-up” approach with patients in the driver seat of this initiative as key to a successful and sustainable achievement. One other initiative that deserves attention is the work conducted at the Arthritis Research UK Primary Care Centre [37]. It has been recruiting lay people since 2006 to constitute a research user group which, like our approach, has capitalized on strategic collaborations with local, regional, national, and international partners to enhance research capacities at their institution. The authors of this publication emphasize the need to build trusting relationships, to adequately organize meetings and working sessions, to provide emotional support and continuous feedback to secure the long-term sustainability of such a patient-oriented hub. Backed by 3 years of achievements in the context of the Province of Québec, the CRCHUS Patient-Partner Committee supports and recommends the need for a dedicated leading and engaged institution to secure the implementation and success of this hub.

Although the real impact of the Committee will be measured more precisely in future years with metrics that will be determined by the Committee together with its partners, its implementation over the last 3 years has made enormous progress on several fronts. Locally, the presence of the Committee, the services that it offers, as well as the added value of engaging patients in research collaborations are increasingly discussed through presentations, conferences and retreats. This dissemination generates positive word-of-mouth sharing of information, resulting in a larger number of researchers that are engaging with patients in collaborations that results in studies that are granted by prestigious funding agencies and managed/co-led by patients that sits on the studies’ Executive Committees. At the level of the province of Québec and nationally, the Committee has contributed its knowledge, know-how, expertise in research and patient engagement to numerous networks, charitable foundations and organizations to secure that research processes and results be more disseminated or better transmitted to the general public as well as to the scientific community.

**Fig. 2:**
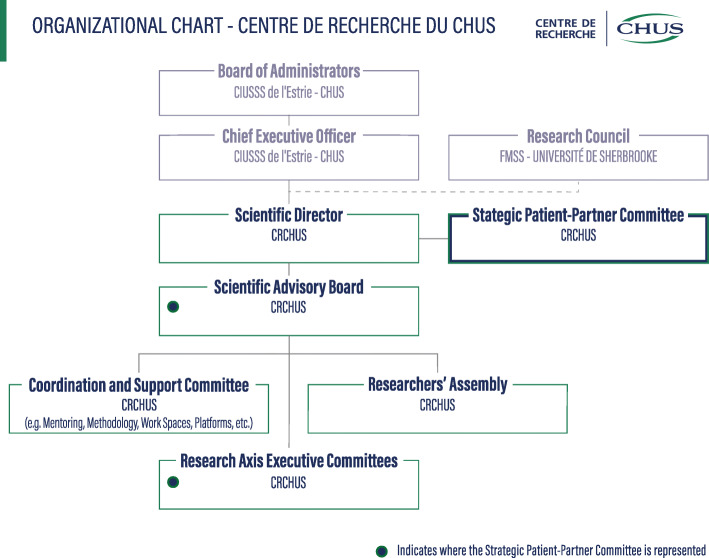
Organization chart – Centre de recherche du CHUS

However, although the Committee contributed to make science accessible to a larger subset of patients and living challenges impregnate a larger number of researchers and partners, it had to overcome one important barrier: the long-standing “imprinting” of a hierarchy that exists between patients and researchers/scientists/physicians. Indeed, while modern societies have idealized medicine, physicians and scientists have paternalized their patients for centuries. Now that patient engagement and co-construction mandates that these two worlds work together, recognizing each other’s expertise is certainly not in their DNA. Slowly but surely, the Committee has overcome barrier through the leadership of the institution and engagement of the committee and its members. Together they ascertain that expectations are well understood, forces discussion, lay transcribe each party’s language. This is also key to recognize each member’s strengths to accomplish the appropriate collaboration and activities with the right partners. One other barrier was to maintain the Committee in constant equilibrium with its evolving ecosystem and be as contextual and relevant as possible. As some actors were reluctant to the integration of the Committee and patient-partners to internal structures and decision-making, baby steps were sometimes necessary. However, the fact that a seat has recently been offered to a patient partner’s representative on the Scientific Council and on the Research Axes Executive Committee of the CRCHUS (refer to Fig. 2 – CRCHUS org chart) demonstrates that the patient-partner’s initiative has full support from its organization.

The upcoming years will challenge the Committee to confirm whether its implementation will have nurtured and maintained a cultural change within and outside the organization. Indeed, the forthcoming phase of the development of the Committee will evaluate the perception of researchers and the decision-makers towards the participation of patients in research (both at the level of projects and governance): determine whether unconvinced and reluctant researchers and decision-makers will have endorsed the approach. Another challenge will be to assess whether the infrastructure created will be sustainable for founding committee member patients to contribute on the longer term. To find strategies to engage new patients, new voices contributing to enlarge the vision of the Committee and that of the partners it represents and works with. The Committee is actually constituted of patient members whom are supporters and positive advocates of research and while they are not representative of the wider population of the Province of Québec, at least half of the patients involved in the Committee are also engaged in other regional, provincial and national Committees. Therefore, this mitigates this bias as they are exposed to the vision and challenges of multiple and diverse patients .

To ensure the generalizability and wider acceptability of our approach, the next step will be to engage patients from under-represented communities as well as patients not involved in research. The last but not the least challenge will be to quantify the Committee’s objectives and impact using strict and context-developed metrics [28, 38]. Indeed, the field of patient-partners is struggling to develop metrics and setting policies to address barriers and secure participation [39]. Accordingly, one of the Committee’s future objectives is to participate in efforts to develop mechanisms to more precisely qualify and, if possible, quantify patient contributions to research under our specific context and based on the PARADIGM framework for monitoring and evaluating patient engagement. We believe that creating a place for patient research partners is essential and that, with the tripartite approach we propose, their systematic participation will be enabled.

We cannot conclude without a comment on financial compensation offered to patient-partners as this is certainly the pain point of many patient involvement set-ups [40, 41]. Volunteer work is certainly something that distinguishes our Committee from other initiatives. While many initiatives encourages that patients be granted financial/salary compensation for their implication, a recent survey highlighted that while less than 8% of patient-partners were motivated by compensation, almost a third were motivated by self-fulfilment (27%) [42]. Accordingly, the CRCHUS Strategic Patient-Partners Committee rather adopted, in its implementation phase at least, a position of sustainability to develop the governance activities: one position that translated in equity between patients and other stakeholders. Unlike some initiatives where 1–2 compensated patients were invited to attend governance meetings with a larger proportion of scientists, the vision of the Committee was to gather as many patients as possible to the same table to enrich the discussions and representativity of the visions. To achieve this goal, it was agreed that all Committee contributors be volunteer, i.e. not receiving any financial compensation other than travel and parking fees. This is also the reason why all meetings were scheduled during after work hours so that all members, including clinicians and institutional members, be volunteering their times and that working patient representatives (one that are often not enrolled due to their poor availabilities during the day) could engage. Committee members also took this decision not to receive financial contribution based on their willingness to freely express their opinions without the conflict of interest related to an employer-employee relationship. While developing the future of the Committee with sustainability of the initiative as a backbone value and acknowledging that at least some research funding agencies have politics that accept compensation for patients involved, and while no compensation policy is yet available for patients involved in the governance of research, the Committee currently is working to present a motion to its institutional decision-makers to recognize the contribution of its actors (equally the patients and the other stakeholders) according to a scale of their involvement. This compensation will favor privileges. Although total financial recognition of their engagement would not contribute to the committee’s sustainability nor freedom of speech; the members are reflecting on different avenues that will permit different forms of recognition without jeopardizing its viability.

## Conclusions

The contribution of patient partners within the CRCHUS organization is welcomed and encouraged. It is obvious that this new alliance builds on human challenges. Our local experience demonstrates that until just recently, patients, researchers and administrators have not had the chance to work as one in developing and advancing the field of research. Now that journals and funding agencies have mandated that these stakeholders work together, they will need to learn how to communicate better and enhance their trust in one another in the co-construction process. Our Committee has developed a local context adapted and sustainable approach to facilitate patient engagement in research governance, expertise and knowledge transfer. Our experience highlights that “basic” human values, such as engagement, respect, transparency, honesty, open minded and generosity, are essential to engage in such an adventure and secure the success of this approach. The endeavour builds on the trust and collaboration established with external partners. Through its vision, the CRCHUS developed an innovative approach and a hub that quickly generated opportunities. Patients, researchers, and administrators must develop a favourable “listening” environment centred on the patient perspective. The increased exchange of heterogeneous knowledge (scientific and experiential) will lead to a greater number of relevant, innovative solutions that will have an impact on science and the health of patients. As the editing of this manuscript was ongoing, the world was struck by the COVID-19 pandemic. This new context does not facilitate the development of new patient-researcher partnerships. However, this never-seen situation has led the Patient-Partner Committee to innovate again and develop new mechanisms to pursue its mission, to influence research (patients recovering from the coronavirus infection, where invited to be part of the reviewing committee of an internal grant competition on COVID-19) as well as to provide patients an opportunity to give a meaning to their health context.

## Abbreviations

CIHR: Canadian Institute of Health Research
CEPPP: Centre of Excellence on Partnership with Patients and the Public
CRCHUS: Centre for Research of the Centre Hospitalier Universitaire de Sherbrooke
DOCC: Diabetes, Obesity and Cardiovascular Complications
PCORI: Patient-Centered Outcomes Research Institute
PPSC: Patient-Partner Strategic Committee
SPOR: Strategy in Patient-Oriented Research
UK: United Kingdom
